# In the skin lesions of patients with mycosis fungoides, the number of MRGPRX2-expressing cells is increased and correlates with mast cell numbers

**DOI:** 10.3389/fimmu.2023.1197821

**Published:** 2023-10-30

**Authors:** Man Hu, Polina Pyatilova, Sabine Altrichter, Caibin Sheng, Nian Liu, Dorothea Terhorst-Molawi, Katharina Lohse, Katharina Ginter, Viktoria Puhl, Marcus Maurer, Martin Metz, Pavel Kolkhir

**Affiliations:** ^1^Institute of Allergology, Charité – Universitätsmedizin Berlin, Corporate Member of Freie Universität Berlin and Humboldt-Universität zu Berlin, Berlin, Germany; ^2^Fraunhofer Institute for Translational Medicine and Pharmacology (ITMP), Immunology and Allergology, Berlin, Germany; ^3^Departement for Dermatology and Venerology, Kepler University Hospital, Linz, Austria; ^4^GV20 Therapeutics, Cambridge, MA, United States; ^5^Department of Dermatology, Heidelberg University, Heidelberg, Germany

**Keywords:** cutaneous T cell lymphoma, mycosis fungoides, pruritus, MRGPRX2, mast cells

## Abstract

**Background:**

Mycosis fungoides (MF) is an indolent T-cell lymphoma that mainly affects the skin and presents with itch in more than half of the patients. Recently, the expression of Mas-related G protein-coupled receptor X2 (MRGPRX2), a receptor of mast cell (MC) responsible for the IgE-independent non-histaminergic itch, has been shown in lesional skin of patients with pruritic skin diseases, including chronic urticaria, prurigo, and mastocytosis. As of yet, limited knowledge exists regarding the MRGPRX2 expression in the skin of patients with MF.

**Objectives:**

To investigate the number of MRGPRX2-expressing (MRGPRX2+) cells in the skin of patients with MF and its correlation with clinical and laboratory characteristics of the disease.

**Methods:**

MRGPRX2 was analyzed in lesional and non-lesional skin of MF patients and healthy skin tissues by immunohistochemistry. Co-localization of MRGPRX2 with the MC marker tryptase was assessed by immunofluorescence. Public single-cell RNAseq data was reanalyzed to identify the MRGPRX2 expression on the distinct cell types.

**Results:**

In lesional skin of MF patients, MRGPRX2+ cell number was higher than in non-lesional skin and healthy control skin (mean:15.12 vs. 6.84 vs. 5.51 cells/mm^2^, p=0.04), and correlated with MC numbers (r=0.73, p=0.02). MC was the primary cell type expressing MRGPRX2 in MF patients. The ratio of MRGPRX2+ MCs to MRGPRX2+ cells in lesional and non-lesional skin correlated with the severity of disease (r=0.71, p=0.02 and r=0.67, p=0.03, respectively).

**Conclusions:**

Our findings point to the role of MRGPRX2 and MC in the pathogenesis of MF that should be investigated in further studies.

## Introduction

Mycosis fungoides (MF) is a type of peripheral non-Hodgkin T-cell lymphoma characterized by its predominant manifestation in the skin. It accounts for 60% of all cutaneous T-cell lymphomas (CTCL) and nearly 50% of all primary cutaneous lymphomas. The disease typically progresses through three stages: patch, plaque, and tumor stage (1). In patients diagnosed with MF, those in the early-stage (stage I or IIA) typically exhibit a relatively indolent disease course. However, individuals with advanced-stage MF (stage IIB or higher) have a considerably poorer prognosis, characterized by a median survival of less than 5 years (2).

MF significantly affects patients’ quality of life (QoL), with itch being one of the most troublesome symptoms (3), occurring in up to 61% of MF patients (4). We have recently reported an association of chronic itch with increased disease severity, a more extensive involvement of the body surface area (BSA), and a pronounced impairment of QoL (5). The mechanisms for itch development in MF patients are not clear. Itch in many MF patients remains refractory to treatment including topical corticosteroids, ultraviolet light, and antihistamines (6).

Recently, Mas-related G protein-coupled receptor X2 (MRGRPX2) has been identified as a mast cell (MC) receptor responsible for IgE-independent MC activation and non-histaminergic itch (7). The MRGPRX2 can cause MCs to release their granules upon binding to a wide range of cationic substances, including neuropeptides, quorum sensing molecules from bacteria, venom peptides, host defense peptides, and FDA-approved drugs (8). Increased numbers of MRGPRX2-expressing (MRGPRX2+) cells have been reported in lesional skin of patients with various skin disorders including mastocytosis (9, 10), chronic urticaria (11) and chronic prurigo (12). As of yet, almost nothing is known about the role and relevance of MRGPRX2 in patients with MF. Here, we investigated the number of MRGPRX2+ cells in the skin of patients with MF and its correlation with itch and other clinical and laboratory characteristics.

## Methods

### Study population

The study was reviewed and approved by the Ethics Committee of the Charité - Universitätsmedizin Berlin (EA4/124/10). The patients/participants provided their written informed consent to participate in this study.

Ten patients with MF (1 female and 9 male, mean age 66.2 years) and 8 healthy controls (3 female and 5 male, mean age: 49.0 years) were included in the study. The patients’ demographic characteristics have been collected and reported in **eTable 1** and elsewhere (5). Briefly, disease severity was assessed by visual analogue scale (VAS), BSA scales, modified severity-weighted assessment tool (mSWAT), and a Likert scale (0–3). Average itch in the last 24h, last week and last month was also assessed by VAS, ranging from 0 (no itch) to 10 (worst imaginable itch). Pruritus‐specific and skin-specific QoL impairments were assessed using Itch-specific quality of life questionnaire (ItchyQol) and the Dermatological Life Quality Index (DLQI), respectively. Patients were also asked about the presence of fatigue, fever, or insomnia. Laboratory parameters, including total serum IgE and serum tryptase levels, were determined at a central laboratory (Labor Berlin GmbH, Berlin, Germany). Eosinophilic cationic protein, major basic protein, IL-31, and substance P were measured using commercial ELISA kits, following the manufacturer’s instructions. Two 6mm diameter skin punch biopsies were taken from MF patients (one from lesional skin and one from non-lesional skin), and one biopsy was taken from healthy controls for histological analysis. In 10 MF patients and 8 healthy controls, biopsies were taken from the upper arm (n=5 and n=7), the trunk (n=3 and n=0), the shoulder (n=2 and n=0) and the lower extremities (n=0 and n=1).

### Histological analysis

MRGPRX2 staining by immunohistochemistry (10 patients and 8 healthy controls) and co-localization of MRGPRX2 with the MC marker tryptase by immunofluorescence (10 patients) were performed as described before (12) (the detailed description is provided in the **Supplementary File** online). Examination of the sections (MRGPRX2 staining and MRGPRX2-tryptase double staining) were carried out using a fluorescence microscope (BZ-X800; Keyence, Itasca, USA). The evaluation of the immunostained sections was done independently and blindly by two experienced investigators. The positive cells were manually counted in at least five horizontally adjacent high-power fields in the upper papillary dermis (for immunohistochemistry staining, ×200, 0.25 mm^2^; for immunofluorescence double staining, ×400, 0.31 mm^2^). Mean values per field were calculated and further converted to “per mm^2^”.

Eosinophil staining was performed as previously described (5). Briefly, wax blocks were cut into 5 µm sections and stained with Giemsa (Merck KG, Darmstadt, Germany) for histology. Two independent and blinded trained investigators counted eosinophils in five or more horizontally adjacent high-power fields (×400, 0.15 mm^2^) in each three layers of papillary dermis, and the average cell numbers per horizontal layer were calculated.

### Single-cell RNAseq data analysis

In order to determine the specific cell types expressing MRGPRX2, we conducted a single-cell analysis of MRGPRX2 expression using publicly available single-cell RNAseq (scRNAseq) data from MF skin tissues (13) and from healthy human skin (14). The scRNAseq data for MF skin tissues were downloaded from the GEO database (accession code: GSE128531), while the healthy human skin data were obtained from cellxgene (Tabula Sapiens). Preprocessing of the scRNAseq data involved normalizing the raw genes counts to the library size, resulting in counts per million. Subsequently, a log transformation was applied to the normalized data. Cell annotation was performed using unknown marker genes.

### Statistical analysis

The statistical analysis was performed using the SciPy (version 1.8.0) in Python 3.9.12. Differences between two independent categories of parametric and non-parametric variables were evaluated using a two-sample t-test or a Mann-Whitney U test, respectively. Differences between three or more independent categories of parametric and non-parametric variables were evaluated using one-way analysis of variance (ANOVA), with a Tukey test used as a *post hoc* analysis, or the Kruskal-Wallis test, with a Dunn test used as a *post hoc* analysis, respectively. The differences between lesional and non-lesional skin biopsy samples were compared using paired t-test or the Wilcoxon signed-rank test for parametric and non-parametric variables, respectively. The correlation between variables was analyzed using Spearman’s rank correlation. Statistical significance was set at P < 0.05. scRNAseq analysis was performed using Scanpy 1.9.3.

## Results

**Figure 1:**
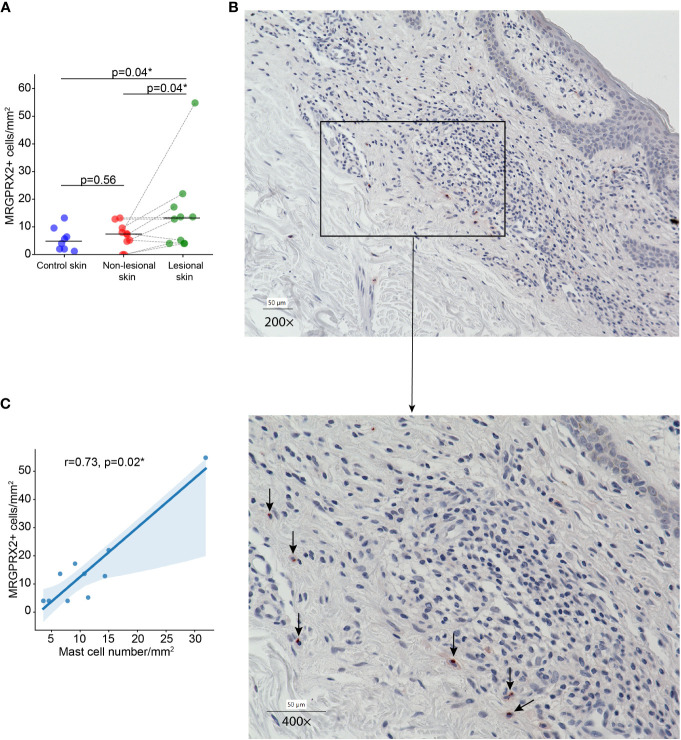
The number of MRGPRX2-expressing cells is increased in lesional skin of MF patients. **(A)** The number of MRGPRX2-expressing cells in lesional and non-lesional skin of MF patients and healthy control skin. **(B)** Immunohistochemical staining of MRGPRX2 in the lesional skin of patient with MF #6, ×200 (above) and ×400 (below) magnification (MRGPRX2+ cells are shown by black arrows). **(C)** Correlation between the number of MRGPRX2-expressing cells and mast cells in lesional skin of MF patients. MF, Mycosis fungoides; MRGPRX2, Mas-related G protein-coupled receptor X2. Mann–Whitney U test and Wilcoxon signed-rank test were used for testing the differences between unpaired data and paired data, respectively. Spearman rank correlation test was used for analyzing the correlation between two independent variables. P < 0.05 considered significant. *p<0.05.

**Figure 2:**
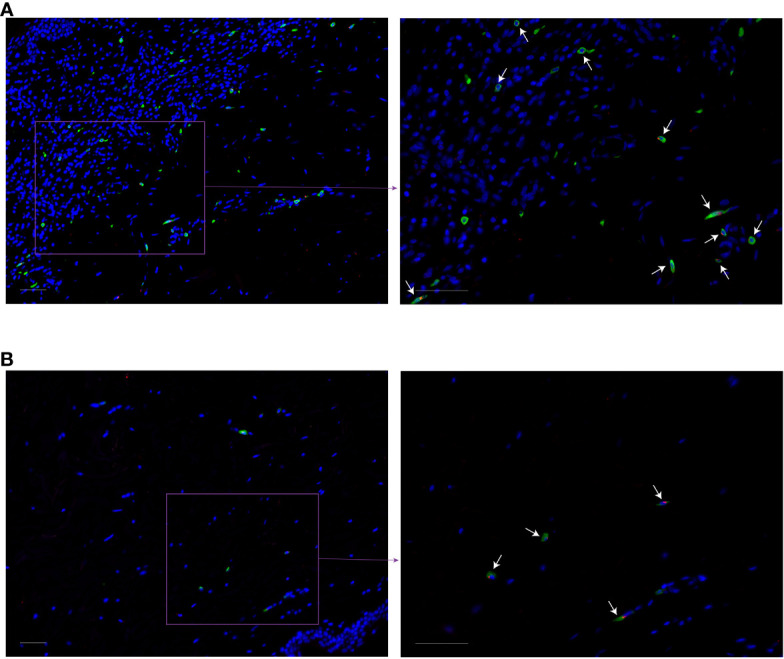
Co-localization of MRGPRX2 with the mast cell marker tryptase in MF patients. Immunofluorescence staining of lesional **(A)** and non-lesional **(B)** skin of the patient with MF #6 with anti-tryptase (green), anti-MRGPRX2 (red), and DAPI (blue). White arrows indicate some of tryptase-MRGPRX2 double-positive cells. ×200 (left) and ×400 (right) magnification. Bar = 50 µm. DAPI, 49-6-diamidino-2-phenylindole dihydrochloride; MF, Mycosis fungoides; MRGPRX2, Mas-related G protein-coupled receptor X2.

**Figure 3:**
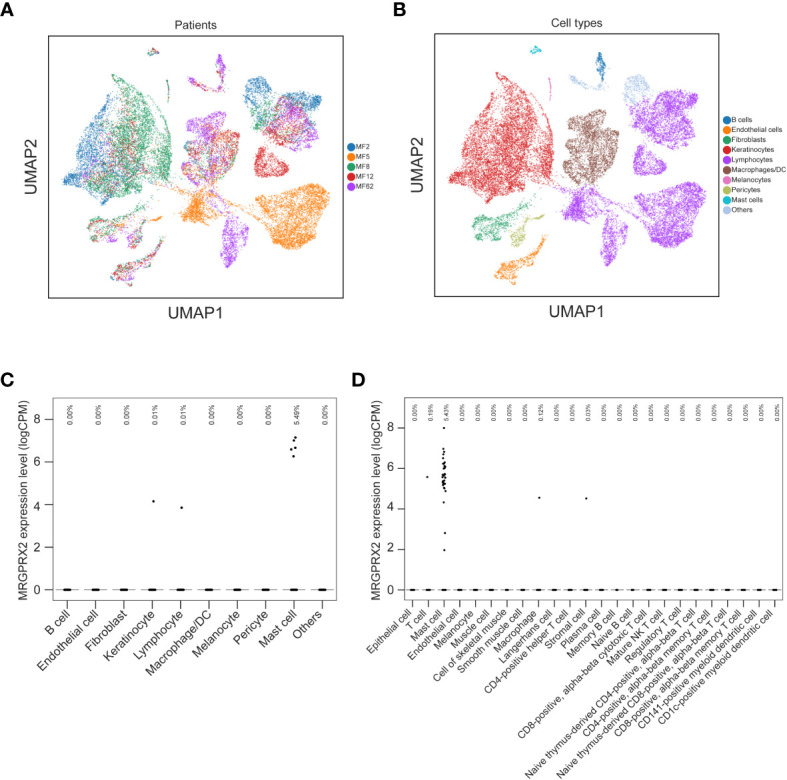
mRNA expression of MRGPRX2 in the skin of MF patients and the healthy skin tissues. UMAPs show the clusters of single cell transcriptomes of skin biopsies from five MF patients **(A)** and the cell types **(B)**. Dot plot illustrates the mRNA expression levels of MRGPRX2 across different cell types in the skin of MF patients **(C)** and in healthy skin tissues from Tabula Sapiens **(D)**. Each dot represents a single cell. Fractions of cells expressing MRGPRX2 are shown at the top of **(C, D)**. MF, Mycosis fungoides; MRGPRX2, Mas-related G protein-coupled receptor X2; UMAP, uniform manifold approximation and projection.

In MF patients, the number of MRGPRX2+ cells in lesional skin was significantly higher compared to non-lesional skin and healthy skin (mean: 15.12 vs. 6.84 vs. 5.51 cells/mm^2^, respectively, p=0.04) (**Figures 1A, B**). The number of MRGPRX2+ cells correlated with MC numbers in lesional (r=0.73, p=0.02) (**Figure 1C**) but not non-lesional skin of MF patients. Double staining for MRGPRX2 and tryptase indicated the co-localization of MRGPRX2 with MCs (**Figure 2**). The number of MRGPRX2+ MCs was higher as compared to MRGPRX2+ non-MCs in both lesional (mean: 6.74 vs. 3.45 cells/mm^2^, p=0.09) and non-lesional skin (mean: 4.04 vs. 2.44 cells/mm^2^, p=0.01). scRNAseq analysis of publicly available data from the skin of MF patients (13) and healthy skin (14) showed that 5.43-5.49% of MCs express MRGPRX2, whereas lymphocytes and keratinocytes showed minimal expression at 0.01% in the skin of MF patients (**Figure 3**). The ratio of MRGPRX2+ MCs to MRGPRX2+ cells in lesional and non-lesional skin correlated with the severity of the disease (r=0.71, p=0.02 and r=0.67, p=0.03, respectively).

**Figure 4:**
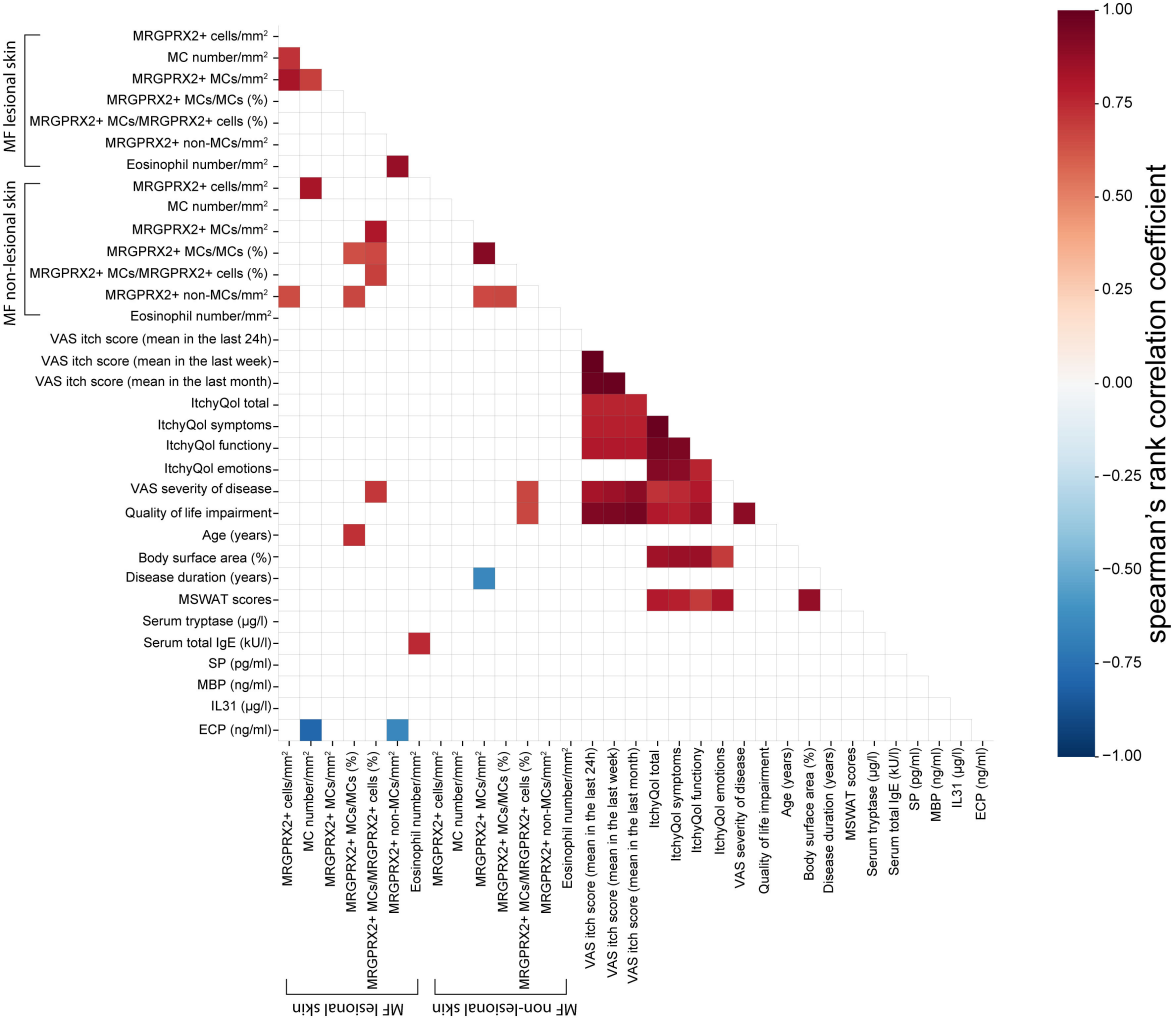
Spearman’s rank correlation matrix between the variables in MF patients. The color of the cells represents correlation coefficients, indicating strength and direction of the correlations, ranging from red (positive correlations) to blue (negative correlations). The strength of the correlation is indicated in the color scale (at the right of the panel). Blank space indicates the correlation was not statistically significant (Spearman correlation with P < 0.05 considered significant). The values refer to valid data only (excluding missing data). ECP, Eosinophilic cationic protein; IL31, Interlukin-31; ItchyQol, Itch-specific quality of life questionnaire; MBP, major basic protein; MC, Mast cell; MF, Mycosis fungoides; MRGPRX2, Mas-related G protein-coupled receptor X2; MSWAT score, Modified severity-weighted assessment tool score; QOL, Quality of life; SP, Substance P; VAS, Visual analogue scale.

MF patients and healthy controls did not differ in terms of gender and total serum IgE levels, although healthy controls were statistically significantly younger than MF patients (**eTable 1**). The age of patients and controls did not correlate with the number of MRGPRX2+ cells or with MC numbers in the skin. Lesional skin and non-lesional skin of MF patients did not significantly differ in numbers of MCs, eosinophils, MRGPRX2+ MCs, the ratio of MRGPRX2+ MCs to MCs, and the ratio of MRGPRX2+ MCs to MRGPRX2+ cells (**eTable 2**). The number of MRGPRX2+ cells did not correlate with disease severity, disease duration, pruritus, QoL impairment, eosinophil numbers, and serum levels of tryptase, total IgE, substance P, IL-31, eosinophilic cationic protein, and major basic protein (**Figure 4**).

## Discussion

This study demonstrates a higher number of MRGPRX2+ cells in lesional skin of MF patients. The increase in lesional MRGPRX2+ cells as compared to non-lesional skin was slightly higher in MF (2.21 times higher, mean: 15.12 vs. 6.84 cells/mm^2^) as compared to previously reported in chronic prurigo (1.50 times higher, mean: 3.98 vs. 2.66 cells/mm^2^) (12) but lower than in indolent systemic mastocytosis (4.29 times higher, median: 22.3 vs. 5.2 cells/mm^2^) (9). Increased numbers of MRGPRX2+ MCs were seen in lesional skin of patients with MC-driven disorders, such as chronic spontaneous urticaria (11), chronic prurigo (12) and cutaneous mastocytosis (10). In line with this, the immunofluorescence analysis and reanalysis of scRNAseq datasets provided further evidence supporting the predominant expression of MRGPRX2 on MCs in both healthy donor skin and MF patients’ skin (13, 14). This finding offers a potential explanation for the positive correlation observed between the number of MRGPRX2+ cells and MCs in lesional skin of MF patients seen in our study.

Although in most MF patients the majority of MRGPRX2+ cells are MCs, other MRGPRX2-expressing cells might be relevant including sensory neurons (15), keratinocytes (15), basophils and eosinophils (16). In patients with indolent systemic mastocytosis, the number of MRGPRX2+ cells correlated with eosinophil number (9). However, we did not see such a correlation in MF patients, and eosinophils were rarely seen in skin samples. As shown by micro-array data, MRGPRX2 is expressed on T cells, which are pathogenic drivers in MF, although not confirmed by real-time PCR and further studies are needed (17). In our scRNAseq data analysis, we could see a small number of T cells expressing MRGPRX2 mRNA.

While the number of MRGPRX2+ cells was elevated, we did not observe any significant correlation with clinical or laboratory characteristics of MF in our patient’s cohort. Similarly, the number of MRGPRX2+ cells did not correlate with the clinical and laboratory characteristics of patients with indolent systemic mastocytosis (9). However, the positive correlation between the ratio of lesional MRGPRX2+ MCs to MRGPRX2+ cells and disease severity points to the clinical relevance of MRGPRX2+ MCs in MF that should be investigated in further studies.

We have not determined skin levels of MRGPRX2 ligands that could account for MCs activation and itch induction. In this context, MRGPRX2 might reflect the development of connective tissue MCs and the number of MRGPRX2+ cells may be less important than the lesional presence of MRGPRX2 agonists, e.g. neuropeptides such as substance P and cortistatin (CST). The serum levels of substance P, an agonist of MRGPRX2, were significantly increased in MF patients and positively correlated with disease severity (18). CST can activate MCs for degranulation and increased numbers of CST-expressing cells and CST-expressing MCs were observed in lesions of chronic prurigo (12). Similarly, CST expression was found in lymphomas and lymphocytic leukemias (17).

Other factors can be responsible for the lack of association between MRGPRX2 and clinical features of MF including altered expression due to receptor internalization and/or genetic polymorphisms (19, 20). Lastly, itch in MF patients might not be dominantly triggered via MRGPRX2 pathway and other mechanisms, e.g. IgE-dependent MCs activation, should be ruled out (21).

The results of our study are limited by the small number of patients. Despite the technology limitations of scRNAseq, which makes it less sensitive to lowly expressed genes like MRGPRX2, the lower detection of MRGPRX2 in scRNAseq does not introduce bias when conducting comparative analysis within the same dataset.

In conclusion, the role and relevance of MRGPRX2, its ligands and MCs in patients with MF need further investigation. Additional studies should include larger patient cohorts and determination of levels of MRGPRX2 ligands in the skin of patients with MF to provide a rationale for MRGPRX2-targeted treatments in this disease.

## Data availability statement

The raw data supporting the conclusions of this article will be made available by the authors, without undue reservation.

## Ethics statement

The studies involving humans were approved by Ethics Committee of the Charité - Universi-tätsmedizin Berlin (EA4/124/10). The studies were conducted in accordance with the local legislation and institutional requirements. The participants provided their written informed consent to participate in this study.

## Author contributions

PK, MMe, and MH designed the study and prepared the manuscript. PK, MMe, MH, PP, and CS analyzed and interpreted the data. PP, MH, and NL performed experiments. SA, DT-M, KL, KG, and VP collected the samples and clinical data. The study was supervised by PK and MMe. All coauthors critically revised and provided substantial input to the manuscript. All authors contributed to the article and approved the submitted version.
