# Supplementary material for: In the skin lesions of patients with mycosis fungoides, the number of MRGPRX2-expressing cells is increased and correlates with mast cell numbers
Source: Front Immunol. 2023 Oct 30;14:1197821. doi: 10.3389/fimmu.2023.1197821 (PMC10646224; doi:10.3389/fimmu.2023.1197821)
Supplement: Supplementary file 1 [file Table_1.docx]

**Histological analysis**

MRGPRX2 staining was conducted using immunohistochemistry, and co-localization of MRGPRX2 with the mast cell marker tryptase was assessed by immunofluorescence, following previously established protocols[1]. Briefly, after embedding in paraffin via standard protocol, sections (5μm) were baked at 60°C for 45 minutes in an incubator. Deparaffinization and rehydration were then carried out using standard procedures. Proteinase K pretreatment was performed for 10 minutes at room temperature.

For immunohistochemistry staining, after a 10-minute room temperature protein block application, sections were incubated with the primary anti-human MRGPRX2 monoclonal antibody (ab167125, Abcam) at 4°C overnight. The following day, a series of steps, including 0.3% H2O2 treatment (5 minutes at room temperature), incubation with polymer-HRP anti-mouse antibody (30 minutes at room temperature), and application of AEC substrate chromogen (20 minutes at room temperature), were performed sequentially with intermediate washing steps. Slides were then rinsed in TBS, counterstained with Mayer’s hematoxylin (ab128990, Abcam), and mounted with an aqueous mounting medium.

For immunofluorescence double staining, after a 10-minute room temperature protein block application, sections were incubated with a mixture of anti-MRGPRX2 (ab167125, Abcam) and anti-tryptase (ab134931, Abcam) antibodies at 4°C overnight. The next day, slides were incubated with Alexa Fluor® 594-AffiniPure goat anti-mouse IgG antibody in TBS and 2% goat normal serum for 30 minutes in the dark at room temperature. Subsequently, after washing, the sections were incubated with Alexa Fluor® 488-AffiniPure goat anti-rabbit IgG antibody, diluted in TBS and 2% goat normal serum, for 30 minutes in the dark at room temperature. Washing steps were carried out with TBS between each incubation. Finally, mounting with a preserving reagent containing DAPI (00-4959-52; Invitrogen) was applied.

| **eTable 1. Demographics and baseline characteristics in mycosis fungoides patients and healthy controls** | | | |  |
| --- | --- | --- | --- | --- |
| Parameter | Patients with MF | Healthy controls | p-value |  |
| Male,n (%)^a^ | 9 (90.00%)* | 5 (62.5%) | p=0.27 |  |
| Age (years), mean ± SD^b^ | 66.2 ± 9.8 | 49.0 ± 14.6 | p=0.01 |  |
| IgE (kU/l), median (IQR)^b^ | 36.6 (18.4-225.8) | 103.9 (25.4-250.0) | p=0.51 |  |
| Abbreviations: IQR, Interquartile range; MF, Mycosis fungoides; n, Number; SD, Standard deviation.  ^a^Chi-square test was used for testing whether two independent unordered binary categorical variables are related to each other.  ^b^Two- sample t test or Mann–Whitney U test was used for testing the differences between two independent categories of parametric and non-parametric variables, respectively.  Mean ± standard deviation (normally distributed data) and median (Interquartile range) (nonnormally distributed data) were shown for numerical variables. Number and percentages were shown for categorical variables.  *Males are generally more often affected by MF, with a male-to-female ratio of 1.6-2.0:1[2]. | | | |  |
|  |  |  |  |  |
|  |  |  |  |  |
|  |  |  |  |  |
|  |  |  |  |  |
|  |  |  |  |  |

| **eTable 2. Co-localization of MRGPRX2 with tryptase in patients with mycosis fungoides** | | | | | | |
| --- | --- | --- | --- | --- | --- | --- |
| Patients # | N of MCs, cells/mm^2^ | N of MRGPRX2+ cells, cells/mm^2^ | N of MRGPRX2+ MCs, cells/mm^2^ | N of MRGRPX2+ non-MC, cells/mm^2^ | Proportion of MRGPRX2+ MCs in MCs, % | Proportion of MRGPRX2+ MCs in MRGPRX2+ cells, % |
| P1L | 7.81 | 2.28 | 1.95 | 0.33 | 25.00 | 85.71 |
| P2L | 9.11 | 10.09 | 5.86 | 4.23 | 64.29 | 58.06 |
| P3L | 10.74 | 9.11 | 4.23 | 4.88 | 39.39 | 46.43 |
| P4L | 4.56 | 5.21 | 2.60 | 2.60 | 57.14 | 50.00 |
| P5L | 11.39 | 7.16 | 2.28 | 4.88 | 20.00 | 31.82 |
| P6L | 31.90 | 34.51 | 27.99 | 6.51 | 87.76 | 81.13 |
| P7L | 14.97 | 8.79 | 7.49 | 1.30 | 50.00 | 85.19 |
| P8L | 14.32 | 13.67 | 7.81 | 5.86 | 54.55 | 57.14 |
| P9L | 6.51 | 6.84 | 4.88 | 1.95 | 75.00 | 71.43 |
| P10L | 3.58 | 4.23 | 2.28 | 1.95 | 63.64 | 53.85 |
| **Mean, lesional skin** | **11.49** | **10.19** | **6.74** | **3.45** | **53.68** | **62.08** |
| P1NL | 8.46 | 4.56 | 3.91 | 0.65 | 46.15 | 85.71 |
| P2NL | 6.51 | 4.56 | 2.60 | 1.95 | 40.00 | 57.14 |
| P3NL | 9.11 | 4.88 | 2.60 | 2.28 | 28.57 | 53.33 |
| P4NL | 5.21 | 3.91 | 2.60 | 1.30 | 50.00 | 66.67 |
| P5NL | 8.46 | 1.95 | 0.65 | 1.30 | 7.69 | 33.33 |
| P6NL | 11.72 | 14.97 | 10.42 | 4.56 | 88.89 | 69.57 |
| P7NL | 8.46 | 8.79 | 5.86 | 2.93 | 69.23 | 66.67 |
| P8NL | 6.51 | 3.91 | 2.60 | 1.30 | 40.00 | 66.67 |
| P9NL | 8.79 | 11.39 | 5.86 | 5.53 | 66.67 | 51.43 |
| P10NL | 6.18 | 5.86 | 3.26 | 2.60 | 52.63 | 55.56 |
| **Mean, non-lesional skin** | **7.94** | **6.48** | **4.04** | **2.44** | **48.98** | **60.61** |
| Abbreviations: L, Lesional skin; MCs, Mast cells; MRGPRX2, Mas-related G protein-coupled receptor X2; N, Number; NL, Non-lesional skin. | | | | | | |
| Co-localization of MRGPRX2 with the mast cell marker tryptase was assessed by immunofluorescence. | | | | | | |

1. Kolkhir, P., et al., *Mast cells, cortistatin, and its receptor, MRGPRX2, are linked to the pathogenesis of chronic prurigo.* J Allergy Clin Immunol, 2022. **149**(6): p. 1998-2009.e5. doi.org/10.1016/j.jaci.2022.02.021

2. Willemze, R., et al., *WHO-EORTC classification for cutaneous lymphomas.* Blood, 2005. **105**(10): p. 3768-85. doi.org/10.1182/blood-2004-09-3502
